# The Dehiscent Facial Nerve Canal

**DOI:** 10.1155/2012/679708

**Published:** 2012-02-21

**Authors:** Sertac Yetiser

**Affiliations:** Department of ORL and HNS, Anadolu Medical Center, Gebze, 41400 Kocaeli, Turkey

## Abstract

Accidental injury to the facial nerve where the bony canal defects are present may result with facial nerve dysfunction during otological surgery. Therefore, it is critical to know the incidence and the type of facial nerve dehiscences in the presence of normal development of the facial canal. The aim of this study is to review the site and the type of such bony defects in 144 patients operated for facial paralysis, myringoplasty, stapedotomy, middle ear exploration for sudden hearing loss, and so forth, other than chronic suppurative otitis media with or without cholesteatoma, middle ear tumors, and anomaly. Correlation of intraoperative findings with preoperative computerized tomography was also analyzed in 35 patients. Conclusively, one out of every 10 surgical cases may have dehiscence of the facial canal which has to be always borne in mind during surgical manipulation of the middle ear. Computerized tomography has some limitations to evaluate the dehiscent facial canal due to high false negative and positive rates.

## 1. Introduction

Facial nerve is the most vulnerable structure in the middle ear during otological surgery. Accidental injury may result with facial nerve dysfunction if the surgeon may not pay enough attention to the site where the bony defects are frequently expected to present. Baxter found that 57% of people have dehiscence of the facial canal in the oval niche [1]. Takahashi and Sando have reviewed 160 temporal bones from 129 individuals and have reported facial canal dehiscences in 74% of them, the most frequent site being at the oval window with a length of 0.4–2.64 mm [2]. Moreano et al. have reviewed 1000 temporal bones and have found 56% incidence of at least one facial canal dehiscence with 76.3% prevalence of bilaterality [3]. The incidence of multiple dehiscences along the course of the fallopian canal in the same temporal bone is much higher in specimens of newborns and young children [4]. Comparative studies of histopathological incidence of facial canal dehiscence are a few. Nomiya et al. have compared 133 temporal bones from 84 otosclerosis cases with 102 normal temporal bones from 70 subjects and have found that the incidence in otosclerosis (49.6%) was lower than normal controls (65.7%) [5]. Di Martino et al. have compared the actual clinical findings in 357 operated cases with 300 temporal bones and have reported fallopian canal dehiscence in 6.4% of the operations and 29.3% of the autopsies [6].

The issue of facial canal anomalies in clinical setting rises some important questions to be solved particularly in medical centers where the training of otologic surgery has routinely been made. The aim of this study is to review the site and the type of such bony defects and variations of the facial canal in patients operated for facial paralysis, myringoplasty, stapedotomy, middle ear exploration for sudden hearing loss, and so forth other than chronic suppurative otitis media with or without cholesteatoma, middle ear tumor, and anomaly.

## 2. Subjects and Methods

Patients who have been operated for otological reasons other than chronic otitis media with or without cholesteatoma in last 4 years were included for the study. Patients' charts, clinical notes, and operation reports were reviewed. Otoscopic findings, type of surgery used, the presence and absence of cholesteatoma, and other intraoperative findings related to the facial nerve were systematically documented. Presence of active or chronic infection with discharging ear, cholesteatoma, middle ear tumors, and middle ear anomaly were the main items for exclusion criteria to rule out the possible erosive effect.

144 patients who have been operated for ear problems in last 4 years were enrolled for the study (48 women, 96 men) with ages ranging from 8 to 66. Of those, 92 were tympanoplasty with or without mastoidectomy, 28 were stapes surgery for otosclerosis, 8 were ossiculoplasty for traumatic injury, 11 were exploration of the middle ear for gradual or sudden hearing loss, and 5 were subtotal facial nerve decompression due to severe paralysis resistive to medical therapy.

Dehiscence of the facial canal was classified in 5 basic groups.

1. If the dehiscence is before the coq, it is classified as *Geniculate ganglion dehiscence. *
2. If the dehiscence is between the second genu and the coq, it is classified as *tympanic or horizontal segment dehiscence. *
3. If the dehiscence is located in the second genu very close to the lateral semicircular canal, it is classified as *dehiscence at the second genu. *
4. If the dehiscence is protruding over the oval window only, it is classified as *dehiscence of oval window niche. *
5. If the dehiscence is after the lower level of the oval window at the mastoid or vertical segment, it is classified as *vertical segment dehiscence. *

On the other hand, in an attempt to qualify the wider dehiscence, if the dehiscence is wide enough extending to both horizontal segment and the second genu, it is classified as second *genu + horizontal segment dehiscence,* and if the dehiscence is extending between the second genu and the inferior level of the oval window, it is classified as *second genu + vertical segment dehiscence*.

Results were also compared with preoperative CT findings in 35 cases only since the preoperative CT was not routine. Thus, it was available only in 25.9% of operated cases (144/35). Fine axial cuts of each CT have been reviewed by an expertise radiologist. If there is a dehiscence, the site has been described as Group-I, no dehiscent; Group-II, suspicious dehiscent; in other words, it is hard to tell whether there is a bony defect around the nerve or not; and Group-III, positive dehiscent.

## 3. Results

**Figure 1:**
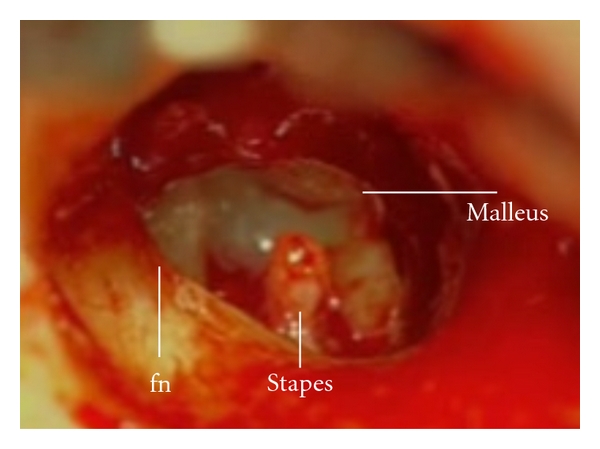
Right ear: middle ear of the patient with incus dislocation after head trauma. Defective facial canal is marked (fn: facial nerve).

**Figure 2:**
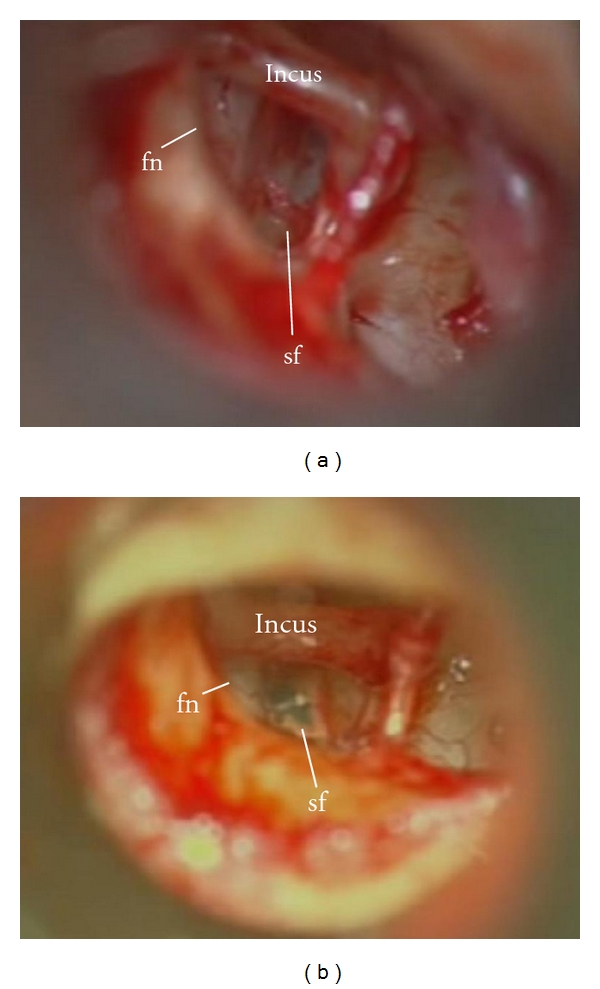
(a) Right ear: middle ear of the patient with otosclerosis. Note the capillary vessel over the facial nerve. (b) Footplate was perforated for insertion of the prosthesis (sf: stapes footplate; fn: facial nerve).

**Figure 3:**
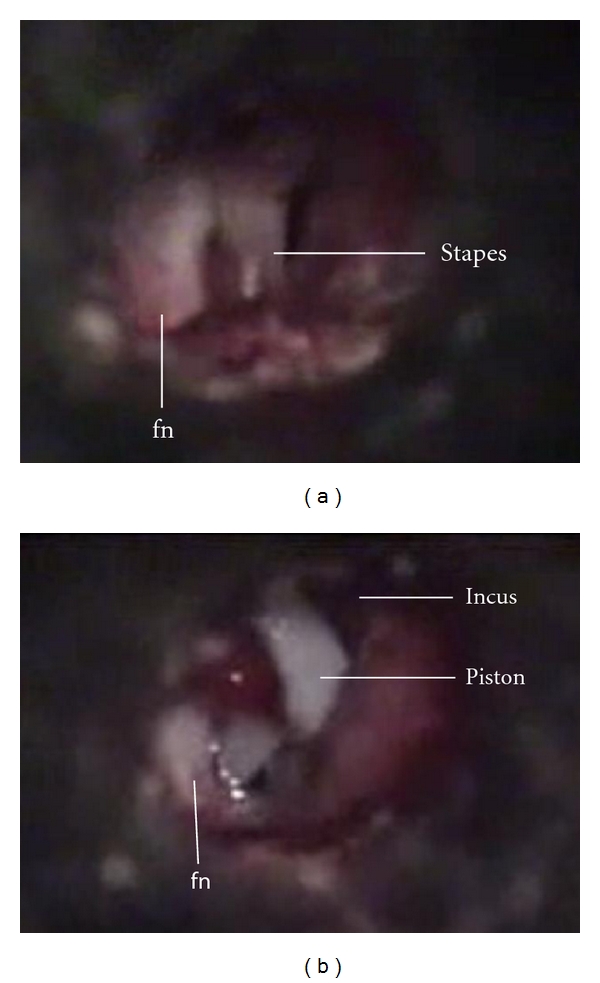
(a) Left ear: middle ear of the patient with otosclerosis. The facial nerve is completely exposed and located anterior to the stapes. (b) The same patient after prosthesis placement (fn: facial nerve).

13 referred ears with tympanic membrane perforation were reoperation due to previous failure. Otherwise all were primary surgery. Intraoperatively, 16 ears (11%) demonstrated an exposed facial nerve. Of those, 5 were at the level of second genu, 4 were at the horizontal segment, 3 were at the level of oval window niche, 3 were at the second genu and horizontal segment, and 1 was at second genu and vertical segment. None of the patients in this series had an isolated geniculate ganglion or vertical segment dehiscence. Of the 16 patients with facial canal defect, 4 were stapes surgery (28/4; 14.2%), 11 were tympanoplasty surgery (92/11; 11,9%), and 1 was middle ear exploration due conductive hearing loss (8/1; 12,5%). This patient had incus dislocation after head trauma without temporal bone fracture (Figure 1). One patient with otosclerosis had completely exposed facial nerve with abnormal course anterior to the stapes (Figures 2(a), 2(b), 3(a), and 3(b)). Four cases demonstrated facial paresis after surgery with minimal cosmetic problem and recovery was uneventful without any intervention in 2 weeks. Demographic data of the patients are presented in Table 1.

**Figure 4:**
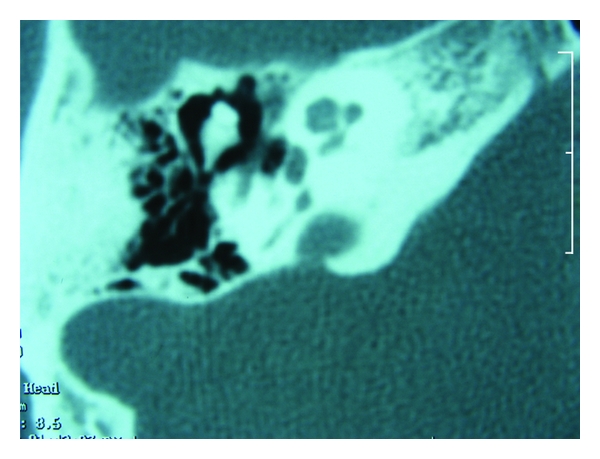
Axial computerized tomography of the patient with otosclerosis. Note that facial nerve is dehiscent at the level of second turn and oval niche.

CT findings have been reported as follows: 19 of 35 cases had no dehiscence (Group-I), it was not certain in 2 (Group-II), and 14 patients had bony defect (Group-III) (Figure 4). Three patients from Group-I and one patient from Group-II had facial canal defect intraoperatively (false negatives according to CT, 21/4 : %19). 13 of 16 cases with intraoperative facial canal dehiscence have been referred to tomography preoperatively. Of those, 9 temporal bone CTs which described dehiscent facial canal preoperatively were correlated with the intraoperative findings. 4 patients did not disclose any facial canal defect (false positive according to CT, 13/4 : %30.7).

## 4. Discussion

 The facial canal is shaped during enchondral ossification of the otic capsule in fetal life. However, it is not completely dependent to the ossification process [7]. Abnormal course of the facial canal is expected in malformed temporal bones and the nerve can be exposed [8]. But, the fibrous layers surrounding the facial nerve seem to be responsible for the final architecture of the facial canal. Therefore, from clinical aspects, it is critical to know the incidence and the type of facial nerve dehiscences in the presence of normal development of the facial canal. The highest incidence of exposed facial nerve has been reported to be 30–35% during surgery for middle ear cholesteatoma [9–11]. Majority of those were found to be in revised cases and at the tympanic segment since it was in the way of extension of the cholesteatoma [12–15]. However, it is difficult to estimate the real number of developmental ones from those due to erosive defect. Patients with tumors, developmental anomalies (atresia), and chronic discharging ears with or without cholesteatoma have been excluded in our series.

It is important to know the nature of such defects to understand the possible underlying mechanism of facial paralysis due to chronic otitis media since a congenital dehiscence or bony defect exposes the nerve to the inflammatory effect of suppuration. Pensak et al. have reviewed 250 consecutive operative cases of chronic otitis media with 54% revision surgeries and have found that an exposed facial nerve was present in 38% of the cases, and of these, 77% of cases had cholesteatoma [16]. Savic and Djeric. have analyzed 64 cases with facial paralysis due to chronic otitis media and reported that the bone destruction of the facial canal is an associated finding in 75% of cases. Tympanic segment was the most common site of involvement (77.2%) which has been stated by the authors that the main reason for this occurrence is the dehiscent facial canal or very thin canal wall most frequently found at this part exposing the nerve to the inflammation [17]. Yetiser et al. have found 83.3% dehiscent facial canal in patients facial paralysis due to chronic otitis media with the most common sites being at second genu and horizontal portions [18]. It is likely true that the bony dehiscence over the nerve is responsible for the extent of the inflammation.

The main group in this series contains patients with otosclerosis. The incidence has been reported to as high as 11.4%–19% [19, 20]. Middle ear has several traps for new beginners to otologic surgery. It is sometimes difficult to identify the facial nerve covered by a thick mucosal layer only. The frequency of iatrogenic injury to the facial nerve has declined with the advent of microsurgical techniques. However, dehiscence at the vestibular surface of the facial canal is usually out of the surgeon's point of view and may be open to an accidental injury. Choung et al. have demonstrated that surgical dehiscence of the facial canal presented in 43% of cases while 73% of those responded to electrical stimulation which indicated an increased vulnerability to trauma and termed this condition as “electrical dehiscence” [21]. Routine monitorization of the facial nerve during surgery of chronic otitis media has been found to be effective and necessary [16, 21].

A high-resolution CT scanning frequently discloses a dehiscence of the bony canal of the facial nerve. However, minor bony defects may stay undetected because of multiplanar and tortuous route of the facial canal. Multiple planes of view are necessary for an optimal image of the canal [22, 23]. Fuse et al. have found that computerized tomography coincided with surgical findings in 75% of cases with 66% sensitivity and 84% specificity [24]. Geniculate ganglion region is particularly important when middle fossa approach is planned. Isaacson and Vrabec have found dehiscent ganglion in 14.5% of 278 cases evaluated by CT scan [25]. Conclusively, one out of every 10 surgical cases may have dehiscence of the facial canal which has to be always borne in mind during surgical manipulation of the middle ear. Computerized tomography has some limitations to evaluate the dehiscent facial canal due to high false negative and positive rates.

## Figures and Tables

**Table 1:** Demographic data of the patients with dehiscent facial nerve canal.

Ear surgery	No. of patients	No. of dehiscent canal	%
Tympanoplasty	92	11	11.9
Stapedotomy	28	4	14.2
FN decompression	5	—	—
Ossiculoplasty	8	1	12.5
ME exploration	11	—	—

TOTAL	144	16	11
